# A Bandpass Filter Realized by Using Pixel Structure and Genetic Algorithm Optimization

**DOI:** 10.3390/mi14071389

**Published:** 2023-07-07

**Authors:** Yangyang He, Yi-Feng Cheng, Jiang Luo

**Affiliations:** 1 School of Electronics and Information Engineering, Hangzhou Dianzi University, Hangzhou 310018, China; heyangyang@hdu.edu.cn (Y.H.); chengyifeng2013@gmail.com (Y.-F.C.); 2 State Key Laboratory of Millimeter Waves, Southeast University, Nanjing 210096, China

**Keywords:** bandpass filter (BPF), genetic algorithm (GA), pixel structure, wideband, EM simulation

## Abstract

This paper presents a flexible method for designing a bandpass filter (BPF) using pixel structure and genetic algorithm (GA) optimization. The pixel structure is made up of a grid of metallic microstrip stubs, and the GA is utilized to determine the connections between these stubs. The pixel structure enables the construction of step impedance and shunt branches, which are used to design a traditional BPF. To enhance the design freedom, one side of the discrete grids is connected to the ground via metallic holes. For verification, a BPF was designed, simulated, and measured. The experimental results showed that the 10 dB return loss bandwidth ranges from 1.1 to 1.9 GHz and the insertion loss is approximately 2.5 dB. There is good agreement between the calculation, EM simulation, and measurement results. The proposed GA-based design method offers significant advantages in terms of one-time EM simulation, feasibility, and labor time savings, making it more convenient than the traditional design method.

## 1. Introduction

In modern wireless communication systems, filters play a crucial role in receiving and transmitting links. Various types of filters, such as multimode resonators (MMRs), step impedance resonators (SIRs), parallel-coupled microstrip lines (PCMLs), split-ring resonators (SRRs), and multi short-circuited stubs, have been developed [1,2]. One such example is the compact quadruple-mode wideband BPF design proposed in [3], which introduces an L-shaped feed-line to achieve a wide bandwidth through four different modes generated by a pair of feed lines. In [4], a compact dual-mode BPF with a half-mode substrate integrated waveguide (HMSIW) cavity is presented, using TE_102_, TE_301_, and TE_101_ modes to create two transmission zeros (TZs) and two poles. Additionally, coplanar strip-line stub resonators are given in [5] to form passband and stopband filters. A novel stopband compact BPF design with an ultra-wide bandwidth based on five-stage SIRs is proposed in [6], which enables the miniaturization and suppression of harmonics. In [7], a dual-band BPF is designed using one zero-value transmission pole (TP), three nonzero TPs, and four transmission zeros. Moreover, ref. [8] demonstrates a high-order dual-port quasi-absorptive BPF composed of a reflective-type coupled-line filter and a quarter-wavelength transmission line, which can remove out-of-band reflections and enhance the roll-off coefficient of the filter. Similarly, ref. [9] presents a quasi-reflectionless microstrip BPF with high out-of-band suppression, utilizing a high-impedance transmission line and a shunt-connected band-stop section. In [10], a novel coupling structure is implemented in a SIW BPF with the modes of TE_101_ and TE_102_, improving the inhibition level of the wide stopband. The resonant unit comprises two identical complementary split-ring resonators (CSRRs) etched on the top surface of the SIW. In [11,12], the stub loaded multiple mode resonator (SL-MMR) technique is employed to design a BPF with a wider bandwidth and better out-of-band rejection performance. However, the aforementioned design techniques are based on the traditional design theory of microwave filters, which usually require complex and time-consuming tuning procedures.

To solve this problem, a novel BPF design technique is proposed, which utilizes pixel structure and GA optimization. The key innovation of this approach is that it requires only a one-time EM simulation, significantly simplifying the design process and saving time. To construct the metal microstrip pixel grid structure, the appropriate number and size of pixel stubs are selected. The GA is then used to identify the optimal connection path in the grid, which is expressed using binary codes 0 and 1. This optimal solution is used to model the BPF in EM-simulation software, and the resulting calculation closely aligns with the simulation results. Finally, a prototype is designed to validate the performance of the proposed BPF.

The paper is organized as follows. In Section 2, the pixel structure and BPF design principle are proposed. And the influence of some key parameters on the optimization results is studied. Subsequently, a novel BPF based on pixel structure and GA is designed and fabricated in Section 3. Finally, the conclusion is described in Section 4.

## 2. Proposed Design Method

**Figure 1:**
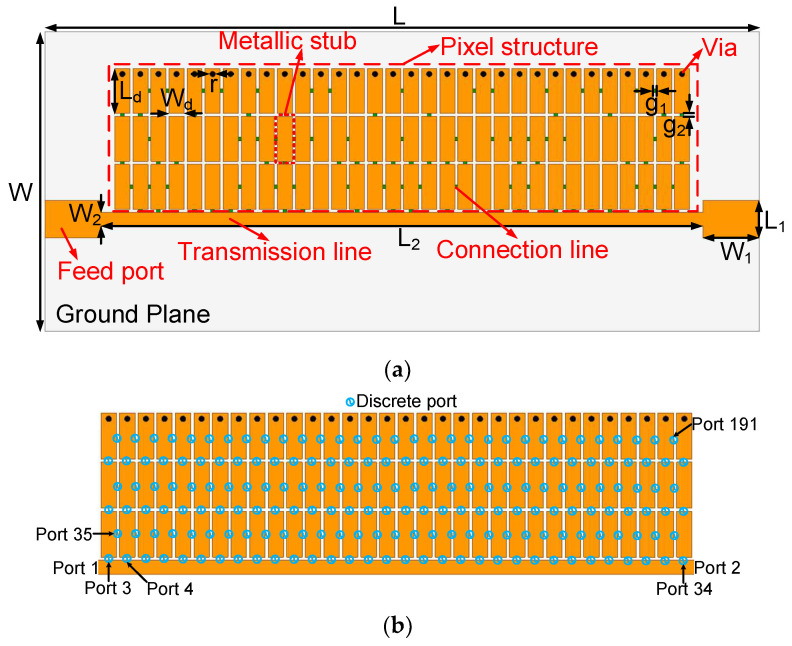
Structure of the proposed BPF: (**a**) layout and (**b**) definition of port numbers. W = 50, L = 90, W_1_ = 5, L_1_ = 3, W_2_ = 1.5, L_2_ = 70, W_d_ = 1.8, L_d_ = 6, g_1_ = 0.2, g_2_ = 0.2, and r = 0.2 (unit: mm).

FR4 with a dielectric constant of 4.4, a loss tangent of 0.02, and a thickness of 0.8 mm was selected to fabricate the proposed pixel structure. Figure 1a shows the layout structure of the proposed BPF, which includes the pixel structure part, the feed line, and the transmission line. The pixel structure consists of a grid of metallic stubs (3 × 32) and some shorting vias. Meanwhile, each metallic stub and connection line have the size of W_d_ × L_d_ and g_1_ × g_2_, respectively. The connection line is inserted between two adjacent stubs, which is determined according to design formulas (described in [13]). In order to achieve the short-circuited microstrip line, one side of the metallic stubs is connected to the ground by shorting vias. The grid number and size can be further expanded when more design freedom is needed.

### 2.1. Design Procedures

An analytical formula was developed to determine where the connection lines should be placed. Figure 1b shows the schematic of adding discrete ports to the pixel structure, which can be regarded as a (2 + 189)-port network. Port 1 and port 2 are external feed ports, and ports 3–191 are internal feed ports. In the simulation, all the ports are set as discrete ports. By running a one-time EM simulation, the Y-parameters of the 191-port network can be obtained. Furthermore, the voltage–current relationship of the network can be written as follows:

(1) $$\left[\begin{array}{c} I_{1}\\ I_{2} \end{array}\right]=\left[\begin{array}{cc} Y_{11} & Y_{12}\\ Y_{21} & Y_{22} \end{array}\right]\;\left[\begin{array}{c} V_{1}\\ V_{2} \end{array}\right]$$

where, *Y*_11_, *Y*_12_, *Y*_21_, and *Y*_22_ are submatrices of the Y-matrix (as illustrated in [13]).

When the connecting conditions are determined, the internal ports will be replaced by corresponding loads while the external port remains unchanged. Then, the 191-port network will become a 2-port network with 189 loads. The diagonal load matrix *Y_L_* is used to indicate the loading of the auxiliary port, and the specific matrix form of *Y_L_* is:

(2) $$Y_{L}=diag\left[y_{1}\;y_{2}\;\cdots \;y_{189}\right]$$

where diagonal elements of *Y_L_* correspond to the load of the auxiliary port.

Using the method introduced in [13], the relationship between the resulting Y-parameters and the different loading conditions can be derived. Here, we use “0” and “∞” to denote short and open connecting conditions, respectively. “0” indicates the presence of a connecting line and “1” represents the condition without a connecting line. Since each internal port has two possible connection states and there are 189 internal ports, therefore, there are 2^189^ different connection schemes. The Y-parameters (*Y_A_*) of the newly obtained 2-port network can be rigorously derived as follows:

(3) $$Y_{A}=Y_{11}-Y_{12}{\left(Y_{L}+Y_{22}\right)}^{-1}Y_{21}$$

Finally, the Y-parameters are converted into S-parameters ([*S_A_*]), and the conversion formula is shown as follows:

(4) $$\left[S_{A}\right]=\left[\begin{array}{cc} s_{11} & s_{12}\\ s_{21} & s_{22} \end{array}\right]=(\left[U\right]-\left[Y_{A}\right])\left(\left[U\right]+{\left[Y\right]}^{-1}\right)$$

where [*U*] represents the identity matrix. Then, the reflection and transmission coefficients are directly obtained. The GA optimization method is used to search for optimal solutions. The design procedure of the proposed BPF is summarized as follows:

(1) Construct the model of the pixel structure with (2 + 189) ports in EM software. Run the simulation to obtain the Y-parameters of the (2 + 189)-port network.
(2) Construct the fitness function of the GA using the obtained Y-parameters and design variables (loading conditions). The binary vector *X* = {*x*_1_, *x*_2_, …, *x*_189_}, *x_i_* ϵ {0,1}, is used to represent the variables during optimization. Referring to [13], the fitness function can be formulated as follows:

(5) $$\underset{X}{\min}\left\{\begin{array}{l} \sum_{k=1}^{k_{1}}\left[w_{21}\times {\left(\left|s_{21}\left(X,f_{k}\right)\right|-t_{21}\right)}^{o}\right]+\sum_{k_{1}}^{k_{2}}\left[w_{11}\times {\left(\left|s_{11}\left(X,f_{k}\right)\right|-t_{11}\right)}^{o}\right]\\ \;+\sum_{k_{2}}^{k}\left[w_{21}\times {\left(\left|s_{21}\left(X,f_{k}\right)\right|-t_{21}\right)}^{o}\right] \end{array}\right\}\;\;\;s.t.x_{i}\in \left(0,1\right)$$

where |*s*_11_ (*X*, *f_k_*)| and |*s*_21_ (*X*, *f_k_*)| represent the reflection and transmission coefficients of the BPF at the considered frequency *f_k_* (*k* is the number of selected frequency points). *k*_1_ and *k*_2_ represent the upper and lower frequencies of the designed filter passband, respectively. *w*_11_/*w*_21_ represent the weighting, and *t*_11_/*t*_21_ are the threshold values. (*a*)° = max (0, *a*) for arbitrary *a* ϵ *R*

(3) Return to steps 1 and 2 when the target results (passband return loss and out-of-band rejection) are not satisfied and increase the design freedom of the pixel structure, such as increasing the number of pixel units or adjusting the size of the pixel units.
(4) When the optimal solution obtained by the GA satisfies the optimization goal, eliminate all the internal ports. Then, according to the resulting optimal solution *X*, “1” indicates the connecting line should be added and “0” means the gap should be reserved. Finally, the layout of the BPF is finished.

### 2.2. Parametric Study

**Figure 2:**
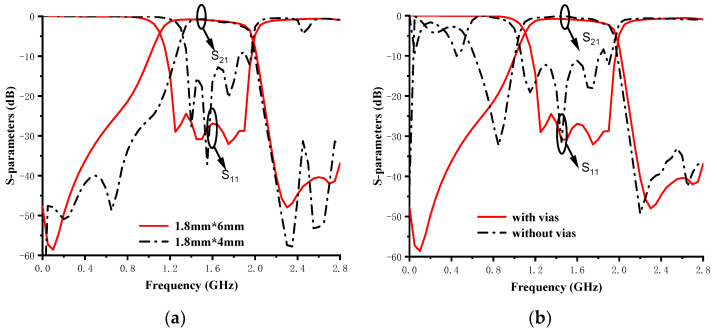
Realized S-parameters using different pixel structures. (**a**) Different sized pixel units and (**b**) with/without shorting vias.

It is worth mentioning that the choice of pixel structure and optimization goal will affect the finally realized performance. Among them, the number of pixel units and the size of the grids are important factors. In addition, shorting some units to the ground can also provide more design freedom. Figure 2 shows the different optimized results using different pixel structures.

Changing the pixel unit size affects the optimal results obtained. As shown in Figure 2a, when the length and width of the pixel elements are adjusted from 1.8 mm × 4 mm to 1.8 mm × 6 mm, the filter performance is significantly improved. Another factor that affects the best optimal solution is whether shorting vias are added to the edge-row pixel units. As shown in Figure 2b, the optimal results with shorting vias are better than that of the structure without vias in terms of out-of-band rejection and passband performance. This is because the design freedom is increased by adding the shorting vias.

**Figure 3:**
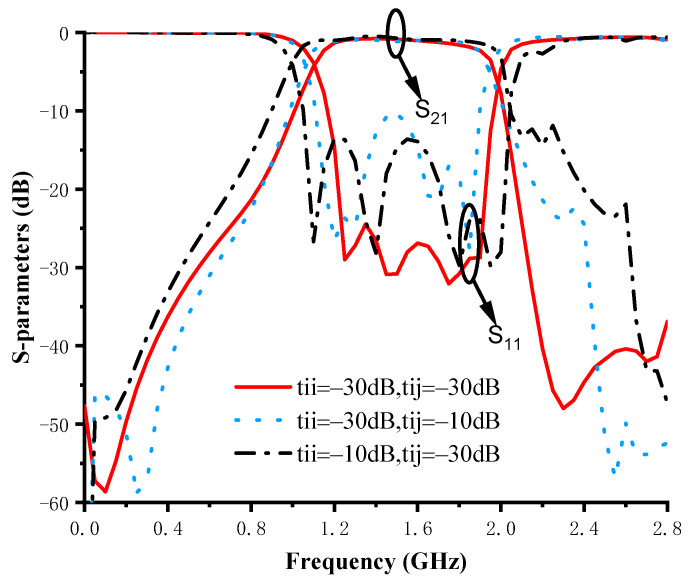
Realized S-parameters of the different optimization targets.

On the other hand, adjusting the optimization target will also affect the optimization result. The solid red line in Figure 3 shows the optimization results obtained by setting the passband return loss threshold *t_ii_* and the out-of-band rejection threshold *t_ij_* to −30 dB. The optimization result obtained with the above optimization target is within the expected range. Then, by adjusting *t_ii_* and *t_ij_* to −10 dB, the blue dashed line and black dotted line shown in Figure 3 are obtained, respectively. Comparing the three different optimization target settings above, it can be found that the optimization target setting also affects the final performance of the designed BPF.

Compared to traditional designs, this proposed method has an attractive feature. One-time EM simulation based on GA optimization can quickly find the optimal solution. Next, the EM simulation and experimental results were utilized to verify the correctness of the directly calculated results.

## 3. Experimental Validation and Results

**Figure 4:**
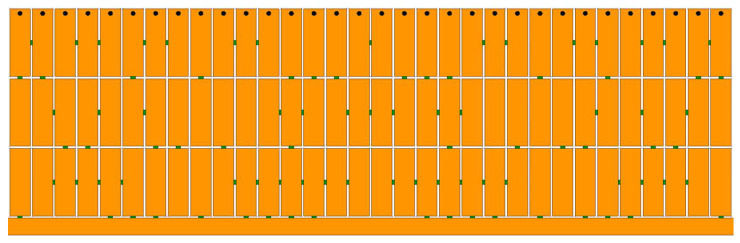
Photograph of the enlarged pixel structure.

**Figure 5:**
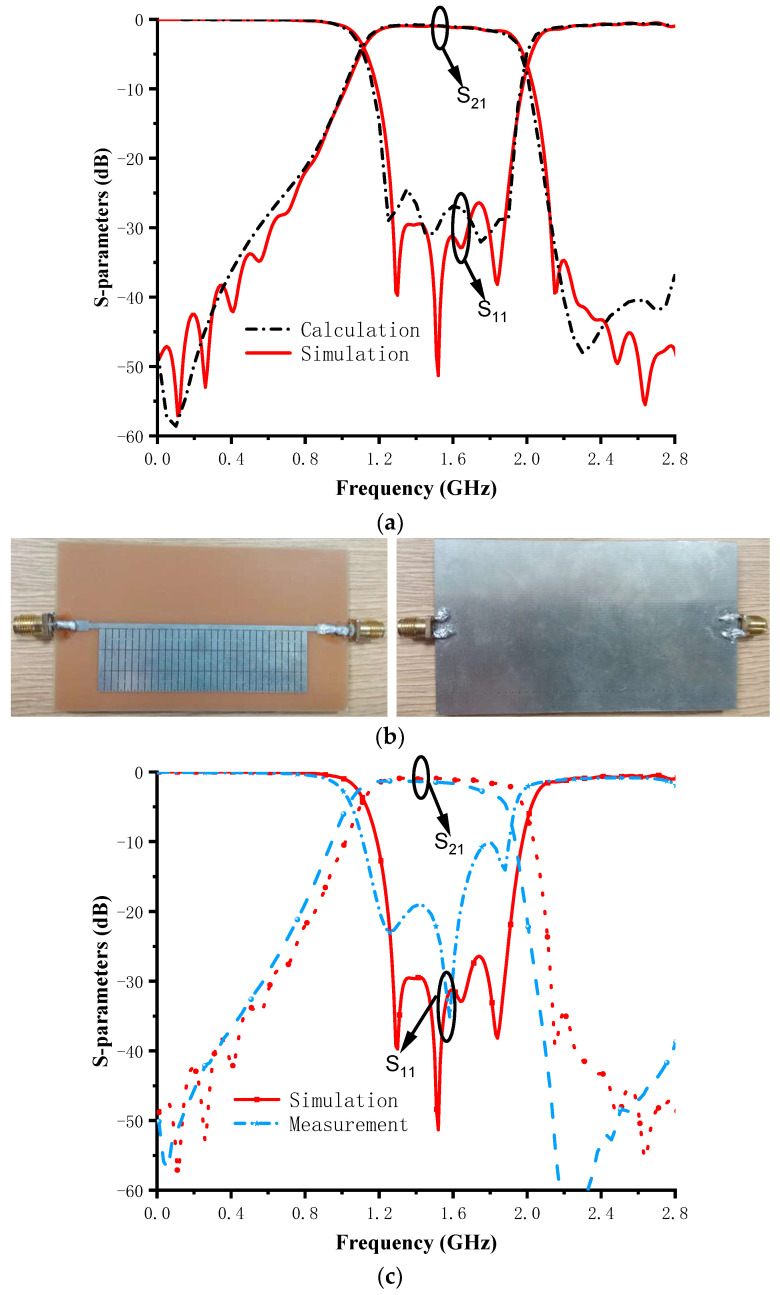
(**a**) Comparison between the calculation and EM simulation results; (**b**) photographs of the fabricated filter; and (**c**) comparison between the simulated and measured S-parameters.

According to the design procedures introduced in Section 2, the designed pixel structure is shown in Figure 4. The small green squares stand for the metallic connection lines, which are built in between two adjacent metallic stubs and between the metallic stubs and the transmission line. The dimensions of the proposed bandpass are listed in Figure 1. In order to obtain the optimal filter performance, the weight (*w*_11_ = *w*_21_ = 1), the total number of frequency points (*k* = 200, uniform sampling from 0.5–2.5 GHz), upper and lower frequencies of the passband (*k*_1_ = 50 corresponds to 1 GHz; *k*_2_ = 150 corresponds to 2 GHz), and the threshold (*t*_11_ = *t*_21_ = −30 dB) of the fitness function were carefully selected. The optimum solution is shown in Table 1; it can be seen that there are 189 binary codes corresponding to the connection status of the metallic connection lines. After obtaining the optimum solution, the proposed BPF was modeled in EM-simulation software. The simulation and calculation results are shown in Figure 5a. Comparing the two results shows a high degree of agreement between them, while a small difference in the out-of-band characteristics still exists. The difference is mainly caused by the connection lines, whose electromagnetic responses are considered in the EM-simulation software but not in the numerical calculation software MATLAB. From the simulation results, it can be seen that the proposed pixel BPF has an operating frequency band from 1.2 to 1.98 GHz with a reflection coefficient under −10 dB. Moreover, the insertion loss is less than 2.2 dB in the operating frequency band.

The theory of GA optimization was proven. Next, a prototype was designed to demonstrate it. The prototype of the fabricated BPF is shown in Figure 5b. The measured results of the proposed BPF are depicted in Figure 5c, the 10 dB return loss (S_11_) bandwidth ranges from 1.1 to 1.9 GHz, and the insertion loss (S_21_) is approximately 2.5 dB. It can be seen that there is a small frequency deviation between the measurement and the EM simulation. The error may be caused by the actual dielectric constant error of the substrate, welding, and the test environment. Meanwhile, there is still consistency between EM simulation and the measurement. Table 2 presents a comparison of the proposed BPF with other recently published designs. This design performs competitively compared to other proposals and does not require tedious EM optimization procedures, shortening the design cycle and saving labor costs.

## 4. Conclusions

This paper proposes a novel method for the fast design of a BPF. The proposed method is based on a 191-ports network, and a GA was utilized to deal with the network to satisfy the designed specification. Through only a one-time calculation, MATLAB will show the optimum binary codes. Based on the calculation codes, connection lines are added or not added between every two metallic stubs. Electromagnetic software was adopted to verify the correctness of the calculation results. Finally, the proposed BPF was fabricated and measured, the measured results show the accuracy of the fabricated BPF. The proposed method based on the pixel structure can save design time and improve efficiency, which determines it to be a good choice in the passive device design field.

## Figures and Tables

**Table 1:** The optimum solution of *X* for port 3 to port 191.

Port Number	Optimal *X*
3~18	0010100110111001
19~34	0101010100011011
35~50	0010001100011101
51~66	1110101111010101
67~82	1001011010000010
83~98	0010110011100111
99~114	0100110111001010
115~130	1010100011011001
131~146	0001100011101111
147~162	0101111010101100
163~178	1011010000010001
179~191	0110011100111

**Table 2:** Comparison between the proposed BPF and other designs.

Ref. No. (Year)	Design Method	Center Frequency (GHz)	−10 dB Bandwidth (%)	Return Loss (dB)	Insertion Loss (dB)	BPF Size (mm^2^)	EM Optimization
[3] 2021	L-shaped feed-line	2.20	38.0	15	0.4	28 × 28	Required
[4] 2021	HMSIW	10.00	5.3	18	2.5	36 × 40	Required
[5] 2018	Coplanar Strip line	5.00	40.0	11	-	3 × 7	Required
[11] 2021	Stub-loaded resonator	3.95	14.0	19	2.4	25 × 50	Required
This Work	GA+ Pixel structure	1.50	53.3	12	2.5	18 × 68	Not Required

## Data Availability

The data presented in this work are available within the article.
